# Laparoscopy in the Surgical Management of Gynecological Cancer: A Comprehensive Update

**DOI:** 10.3390/jcm14217614

**Published:** 2025-10-27

**Authors:** Stamatios Petousis, Georgia Margioula-Siarkou, Chrysoula Margioula-Siarkou, Aristarchos Almperis, Frederic Guyon, Konstantinos Dinas

**Affiliations:** 1 Gynaecologic Oncology Unit, 2nd Department of Obstetrics and Gyanecology, Aristotle University of Thessaloniki, 54624 Thessaloniki, Greece; 2 Institut Bergonie, 33076 Bordeaux, France

**Keywords:** laparoscopy, gynecological cancer, ovarian cancer, endometrial cancer, cervical cancer, vulvar cancer, breast cancer

## Abstract

A laparoscopic approach has been incorporated into the surgical management of a great variety of gynecologic pathologies during the decades following the first description of the method. As knowledge and experience about the use of laparoscopy is accumulating, it is gradually being recognized as an oncologically safe and effective option for the surgical management of various types of gynecological cancer, and the indications for its applications are increasing, as controversial topics are resolved through research. Endometrial cancer is the gynecological malignancy with the most straightforward indications of laparoscopy in its treatment, since a minimally invasive approach is considered the standard of care for both the surgical treatment of early-stage disease and surgical staging through sentinel lymph node biopsy. The role of laparoscopy was significantly decreased in the surgical management of cervical cancer after the publication of the LACC trial which reported worse survival outcomes for patients treated with laparoscopy, and laparotomy has emerged as the preferred approach. However, laparoscopy can be acceptable for carefully selected cases of early-stage cervical cancer and has also been introduced as an effective method for the surgical staging of the disease. The use of laparoscopy in the diagnostic and therapeutic management of ovarian cancer is not fully established but is receiving growing attention, as increasing evidence supports the safety of this approach, especially in the treatment of early-stage disease, where it is considered an acceptable alternative approach to laparotomy. Finally, as laparoscopic advancements are continuously achieved, new indications for laparoscopy have been explored for both vulvar and breast cancer. Future research will identify and highlight new ways to further integrate laparoscopy into the diagnostic and therapeutic management of gynecological malignancies.

## 1. Introduction

Gynecological cancer, defined as any type of malignancy originating from the female reproductive system, is the most common type of cancer and a prominent cause of cancer-related death in females worldwide [1,2]. The increasing prevalence of gynecological cancers has further accentuated the importance of incorporating novel techniques into the therapeutic management of gynecologic oncological cases, in an effort to provide effective treatments that improve not only survival outcomes but also quality of life [3,4]. Under this scope, the role of laparoscopy has been expanded in previous years in the surgical management of gynecological malignancies.

Laparoscopic surgery (LPS), introduced in 1901 by the surgeon and gastroenterologist Georg Kelling, who was the first doctor to insufflate with air and inspect, with the use of a Nitze cystoscope, the abdominal cavity of a dog, was eventually recognized as a groundbreaking innovation which rapidly developed during the following decades [5]. Laparoscopy has been established as an approach across surgical specialties, and operations of increasing complexity have been performed as the technique evolved throughout the years, with continuous and tremendous advancements in terms of its equipment and techniques [5]. However, laparoscopy as a diagnostic technique, which dominated most of the 20th century, must be distinguished from LPS, which emerged as a therapeutic intervention in the 1980s with the advent of video-laparoscopy and the first laparoscopic cholecystectomies [5]. Since then, continuous advancements and techniques have rapidly evolved LPS, enabling its usage in increasingly complex operations across surgical fields [5].

The advantages of LPS over traditional laparotomy are hardly controversial, including consistently faster postoperative recovery, shorter hospital stays, less blood loss, and reduced pain, complication rates, and perioperative morbidity [6,7]. As a result, the laparoscopic approach has been widely adopted in gynecological practice for both benign and malignant conditions [8,9]. Current evidence also strongly supports LPS as both an oncologically safe and effective option for certain types of gynecological cancer [10].

However, there are legitimate concerns around LPS in gynecologic oncology, including a surgeon’s experience and learning curve, the risks of tumor spillage, and port-site metastases, all of which can affect the oncological outcomes compared to open surgery. Recent multicenter randomized controlled trials (RCTs), most notably the Laparoscopic Approach to Cervical Cancer (LACC) trial, raised serious doubts about the oncological safety of minimally invasive techniques in particular clinical situations, demonstrating how challenging it is to conduct well-designed multicenter trials in surgical oncology [10,11].

The objective of this comprehensive review is to efficaciously summarize and combine cumulative knowledge and the up-to-date evidence about the role of laparoscopy in the surgical treatment of gynecological cancers, in terms of indications, techniques, and patients’ outcomes, as well as to explore and describe novel and developing techniques in this field.

## 2. Endometrial Cancer

Over the course of the last few decades, a gradual increase has been observed in the use of laparoscopy for the surgical management of endometrial cancer (EC) [12]. Nowadays, EC is the main gynecological malignancy for which the laparoscopic approach is widely accepted and implemented in clinical practice. The disease is classified according to the depth of myometrial invasion (stage IA: <50% invasion; stage IB: ≥50% invasion) and the histopathological type (endometrioid vs. non-endometrioid subtypes including serous, clear cell, carcinosarcoma, and undifferentiated carcinoma), which together determine prognosis and guide therapeutic decisions [13].

### 2.1. Early-Stage Endometrial Cancer

The majority of patients with EC receive their diagnosis in the early stages of the disease, due to an early onset of symptoms, with vaginal bleeding usually being the first clinical manifestation, especially in postmenopausal women [14]. Surgery is the main treatment offered in women with early-stage EC [15]. The oncological safety of LPS in the therapeutic management of early-stage EC has been the objective of intensive research efforts over previous years and has been established through randomized clinical trials to offer equivalent survival with better perioperative outcomes [16,17]. Janda et al. reported that, for stage I endometrioid EC, disease-free and overall survival and recurrence rates did not significantly differ between patients that underwent laparoscopic and abdominal total hysterectomy [16]. Similarly, the Gynecologic Oncology Group LAP2 study compared survival and recurrence rates in women with early EC treated with laparoscopy versus laparotomy and reported similar survival outcomes for both approaches, with the laparoscopic approach proven to be superior in terms of short-term safety and length of hospital stay [17]. These conclusions were further validated by meta-analyses, which highlighted the reduced perioperative morbidity, blood loss, and length of hospital stay as advantages of laparoscopic surgical treatment [12,18]. Since strong evidence has been acquired, both ESGO guidelines and ISGE guidelines recommend minimally invasive surgery as the gold standard and surgical approach of choice for early-stage EC, including for women with high-risk endometrial carcinoma [13,19]. The standard surgical procedure is a total hysterectomy with bilateral salpingo-oophorectomy, preferably using a minimally invasive method, without vaginal cuff resection or parametrectomy. The bilateral removal of tubes and ovaries is standard because EC can metastasize to the ovaries and shares hormonal risk factors with ovarian cancer (OC). Staging infracolic omentectomy is mandatory in clinical stage I serous carcinoma, carcinosarcoma, and undifferentiated carcinoma due to their propensity for peritoneal spread. It is not necessary for stage I endometrioid or clear cell carcinoma [13]. For patients with high–intermediate-risk/high-risk EC that have undergone surgical treatment with incomplete staging, surgical restaging can be considered in cases where the outcome might influence the design of the adjuvant treatment strategy [13]. General principles for the laparoscopic surgical management of early EC include the avoidance of intraperitoneal tumor spillage and morcellation of the surgical specimens. The identification of extra-uterine metastatic disease, apart from nodal involvement, presents relative contra-indications for the laparoscopic approach, since extensive cytoreductive surgery may require laparotomy. In clinical practice, if metastatic disease is discovered intraoperatively during laparoscopy, the decision to convert to laparotomy depends on the extent of disease, the surgeon’s laparoscopic expertise, and whether complete resection is achievable. Isolated peritoneal deposits or small-volume disease may be managed laparoscopically by experienced surgeons [13]. Fertility-sparing surgery with preservation of the ovaries is an acceptable choice in premenopausal patients aged <45 years without cancer family history involving OC risk, diagnosed with low-grade endometrioid endometrial carcinoma with myometrial invasion < 50% and no metastatic disease in the ovaries or outside the uterus and the cervix, but salpingectomy is required in these cases [13,20]. Moreover, uterus-sparing treatment can be offered in patients with grade 1 endometrioid endometrial carcinoma, stage IA, without myometrial invasion and without risk factors, desiring future pregnancy. In this case, according to the ESGO-ESHRE-ESGE guidelines, the most effective fertility-preserving approach is a hysteroscopic tumor resection, followed by oral progestins and/or a levonorgestrel intrauterine device [20].

### 2.2. Surgical Staging

The surgical staging and assessment of nodal status is necessary to accurately identify the stage of EC and further tailor the treatment strategy, considering that the presence of nodal metastasis is a major prognostic factor and a criterion significantly affecting the decision for the administration of adjuvant therapy [21]. Although systematic lymph node dissection has been the gold standard surgical staging procedure in the past, a noticeable shift to sentinel lymph node (SLN) mapping has occurred in recent years, in an effort to minimize and avoid the morbidity and complications following lymphadenectomy [22]. Through various studies, as well as meta-analyses, SLN biopsy has been recognized as an accurate and effective method for the evaluation of nodal involvement in EC [21,23,24]. According to ESGO guidelines, laparoscopic SLN biopsy is recommended as a staging procedure in women with early-stage, low-risk/intermediate-risk EC. Additionally, when myometrial invasion is not identified in this category of patients, through preoperative imaging, SLN mapping can be omitted [13]. Regarding the mapping technique, the use of near-infrared fluorescent laparoscopic equipment and the intracervical administration of indocyanine green (ICG) are considered the standard of care, with increased detection rates compared to other tracers [13,25]. Regarding patients with high–intermediate-risk/high-risk disease, systematic lymph node staging should be performed as the standard of care. However, the indications of SLN biopsy have recently been expanded, potentially including nodal assessment in patients with early-stage, high–intermediate-risk/high-risk EC. With available evidence from an increasing number of studies supporting that SLN biopsy could be performed in high-risk EC cases with acceptable detection rates and diagnostic accuracy, replacing systematic lymphadenectomy [26,27,28,29], the ESGO guidelines suggest that SLN identification and excision is an acceptable alternative to systematic lymph node dissection, which remains the standard of care for lymph node staging in stage I and II high–intermediate-risk and high-risk EC [13]. Nevertheless, more prospective studies are required to solidify the implementation of SLN mapping as standard practice for patients with high-risk EC. Although gradually losing ground, when indicated for early-stage EC, systematic lymphadenectomy can be safely performed via the laparoscopic approach by trained and experienced gynecologic oncologists [30].

### 2.3. Advanced-Stage Endometrial Cancer

The management of advanced-stage EC significantly depends on the assessment of resectability of the disease, as it is determined by full preoperative staging and discussion in a multidisciplinary tumor board. Per ESGO guidelines, systemic therapy is advised and preferred in case upfront surgery is not achievable, but an extensive debulking surgery, with the inclusion of enlarged lymph node dissection, should be discussed and offered to patients with stage III and IV EC, in case complete macroscopic resection can be achieved, with acceptable morbidity and postoperative quality of life [18]. In this setting, a minimally invasive surgery could be considered an acceptable approach for carefully selected patients [13,31]. Despite recent evidence supporting that the laparoscopic surgical management of advanced-stage EC has similar, if not better, perioperative results compared to laparotomy, without impairing survival [31,32], these data are limited and not sufficient to fully establish the implementation of a laparoscopic approach as standard practice for this category of patients.

#### Key Points—Summary of Current Clinical Practice

The minimally invasive approach is the standard of care for the surgical treatment of early-stage EC, even in cases of high-risk endometrial malignancies. A laparoscopic total hysterectomy with bilateral salpingo-oophorectomy should be performed in these patients, with the potential addition of infracolic omentectomy, based on the histological subtype of the EC, while a fertility-sparing surgery can be offered under conditions in younger patients, with preservation of the ovaries or/and the uterus. Surgical nodal assessment is necessary to accurately define the stage of the disease; laparoscopic SLN mapping with the intracervical injection of ICG is the staging procedure of choice for women with early-stage, low-risk/intermediate-risk EC and is considered an acceptable alternative procedure of nodal assessment for cases of stage I and II high–intermediate-risk and high-risk EC, in which (laparoscopic) pelvic systematic lymphadenectomy remains the gold standard. For operable advanced-stage EC, abdominal debulking surgery is recommended, and a laparoscopic approach is reserved, due to the lack of sufficient evidence, only for carefully selected patients.

## 3. Cervical Cancer

While the role of laparoscopy gradually but steadily expands in the management of the majority of gynecological malignancies, it was, on the contrary, fundamentally questioned and eventually decreased in patients with cervical cancer during the last few years.

### 3.1. Early-Stage Cervical Cancer

The management of cervical cancer significantly differs between patients with early- and advanced-stage disease, as surgical treatment is feasible for patients with up to IB2 stage and IIA2 stage, but chemoradiation is the treatment of choice for patients surpassing this stage, since the general principle of the treatment strategy for cervical cancer is to avoid the combination of radical surgery and radiotherapy due to the increased morbidity of the combined treatment [33]. Although laparotomy has been the standard of care, minimally invasive surgery, either laparoscopic or robotic-assisted, was reintroduced into the surgical treatment of cervical cancer in the mid- and late 2000s [34]. This shift to the laparoscopic approach was suddenly overthrown after the publication of the Laparoscopic Approach to Cervical Cancer (LACC) trial in 2018 by Ramirez et al., a prospective clinical trial designed to compare survival outcomes between patients with early-stage cervical cancer treated with minimally invasive and abdominal radical hysterectomy [11]. The study concluded that minimally invasive radical hysterectomy was followed by a significant decrease in disease-free survival and overall survival rates compared to abdominal radical hysterectomy [11]. These results had such a great impact on clinical practice that the laparoscopic approach was nearly abandoned for the surgical treatment of early-stage cervical cancer [35]. In an effort to explain these results, various hypotheses have been formulated, including regarding the type of minimally invasive surgery, the size of the cervical lesion, and the use of uterine manipulators and protective measures, which were later thoroughly investigated in subsequent studies [36]. The SUCCOR trial, published in 2020, shed some light on the potential causes that may explain the poorer oncological outcomes of LPS that the LACC trial reported. Chiva et al. reported that the avoidance of uterine manipulators and the implementation of surgical maneuvers such as preoperative vaginal closure that reduced tumor spillage during colpotomy in minimally invasive surgery were found to have similar oncological outcomes to open surgery for patients with stage IB1 cervical cancer who underwent radical hysterectomy [37]. Various scientific efforts have since been dedicated to further interpreting and assessing the outcome of the LACC trial and big multicenter retrospective studies, such as the 4C (Canadian Cervical Cancer Collaborative) study and MEMORY study, reporting that the laparoscopic approach does not appear to compromise oncological outcomes when compared to the open surgical route for women with early-stage cervical cancer [38,39]. At the same time, prospective studies have been designed and launched to re-evaluate the role of minimally invasive surgery in the surgical management of cervical cancer and potentially provide evidence that could support its safe reintroduction into clinical practice, such as the robot-assisted approach to cervical cancer (RACC) trial, which is estimated to close in May 2027 [40]. According to current ESGO guidelines, laparotomy is the approach of choice when surgical treatment is required, including radical parametrectomy, but a minimally invasive approach can be considered solely in cases of low-risk tumors (<2 cm and free margins after conization), treated in centers experienced in performing radical hysterectomy with minimally invasive surgery, which adhere to the ESGO quality criteria for surgery, and only when the patient consents after detailed discussion about current evidence [32].

### 3.2. Surgical Staging

Following the principles of surgical staging as they were previously described for EC, it is of utmost importance to surgically assess the nodal status of patients with cervical cancer in order to determine the stage of the disease correctly and accurately [41]. Apart from being a major prognostic factor, nodal involvement can also fundamentally alter the treatment plan and dictate the need for adjuvant therapy [42,43]. The histological identification of a sole metastatic pelvic (or para-aortic) lymph node upstages the disease to stage IIIC cervical cancer, meaning that surgical treatment is no longer an eligible option [44,45]. For early-stage cervical cancer, the ESGO guidelines recommend that lymph node evaluation should precede any other surgical maneuver, while the laparoscopic approach is considered acceptable for any procedure regarding nodal assessment. SLN biopsy should be performed, with ICG as a tracer, and the use of near-infrared fluorescent laparoscopic imaging and evaluation of the excised nodes by frozen section is recommended. In case nodal metastasis is histologically confirmed, any further surgical treatment is not feasible, and the patient should be referred for definitive chemoradiotherapy. In the case of negative SLNs, systematic pelvic lymphadenectomy should be performed bilaterally as a standard nodal staging procedure, while para-aortic lymphadenectomy at least up to the inferior mesenteric artery can also be performed for staging purposes [32].

Surgical nodal assessment has been established as common clinical practice for early-stage cervical cancer, but recently it has also been gaining interest as a nodal staging procedure for cases of locally advanced cervical cancer as an alternative to imaging staging. Locally advanced cervical cancer is associated with a higher incidence of nodal metastasis, which affects not only the patients’ staging, but also the development of radiotherapy scope [46]. Although radiological staging is the conventional method to determine nodal metastases, there are limitations of imaging that should be considered, especially in determining whether para-aortic lymph nodes are metastatic [47]. Positron emission tomography/computed tomography (PET/CT) is unable to identify approximately 25% of metastases at the aortic lymph nodes, which presents rather important information for the design and mainly the determination of the field of radiotherapy [48]. While a variety of studies support that surgical staging, especially when performed laparoscopically, is highly accurate for determining lymph node metastasis, has only a few associated complications and can reduce complications associated with extended field radiotherapy [49,50], it has not yet been established as the standard of care. The current ESGO guidelines recommend PET CT as the standard method for nodal assessment in patients with locally advanced cervical cancer, with pelvic and para-aortic lymphadenectomy at least up to the inferior mesenteric artery noted as an acceptable alternative approach, which acquires a rather significant role when imaging is indicative of metastatic pelvic lymph nodes with negative para-aortic nodes, as it can provide definitive answers that will determine the need for elective para-aortic external beam radiotherapy [32]. At the moment, the PAROLA trial, a multicenter, randomized, phase III study, has been launched with the objective of investigating whether survival outcomes after chemoradiation for locally advanced cervical cancer significantly differ between patients who have been staged surgically with para-aortic lymphadenectomy or radiologically with PET CT. The results of this trial are expected in 2027 and could potentially change the clinical practice regarding standard staging procedures [51].

#### Key Points—Summary of Current Clinical Practice

After the publication of the LACC trial, which significantly influenced the guidelines issued about the surgical treatment of operable cervical cancer (up to stage IB2 and IIA2), abdominal radical hysterectomy was reinstated as the gold standard. The minimally invasive approach is only acceptable for patients with low-risk tumors (<2 cm and free margins after conization) who are being treated in centers experienced in performing laparoscopic radical hysterectomy. Surgical staging with a pelvic SLN biopsy should always precede in cases of operable cervical cancer and can be performed laparoscopically, followed by a systematic pelvic lymphadenectomy in cases of negative frozen section of the SLNs, while further surgical treatment is aborted if the SLNs are positive. For the appropriate staging of suspected locally advanced cervical cancer, PET-CT remains the recommended staging modality, but considering that it may fail in recognizing metastatic lymph nodes in a significant percentage of cervical cancer patients, (laparoscopic) pelvic and para-aortic lymphadenectomy at least up to the inferior mesenteric artery is an acceptable alternative staging approach.

## 4. Ovarian Cancer

The use of laparoscopy in the diagnostic and therapeutic management of OC is controversial but is gradually receiving attention and acceptance in surgical practice, as increasing evidence supports the oncological safety of this approach in cases where it is indicated both in early- and advanced-stages of the disease.

### 4.1. Suspicious-for-Malignancy Ovarian Masses

Although laparoscopy is considered the gold standard for the excision of benign adnexal neoplasms, the laparoscopic management of adnexal masses suspicious for malignancy has been a controversial topic [52,53]. The main concerns regarding the use of the technique are the intraoperative puncture and rupture of the suspicious mass, the potential subsequent dissemination of cancerous cells in the peritoneal cavity and upstaging from stage IA to IC, and port-site metastases, while the size of the neoplasm is another factor creating controversy, since laparoscopy was not initially considered feasible for large masses [52,53,54,55]. However, these concerns are gradually resolving, and the laparoscopic surgical resection and staging of suspicious adnexal lesions is nowadays considered a safe alternative, but under certain conditions. In the case of a high suspicion of malignancy according to preoperative testing, a referral to a specialized department with a gynecologic oncologist is suggested [53,56]. Regarding the surgical procedure itself, en bloc oophorectomy or adnexectomy is preferred in the case of a high suspicion of malignancy instead of cystectomy, and techniques to avoid rupture of the mass and spillage should be applied, such as the use of laparoscopic watertight endobags that can be removed through a 10 to 15 mm port [57,58,59]. Moreover, it is important to perform adequate complementary surgical staging in cases of a high suspicion of malignancy or when early-stage cancer is incidentally detected during surgery for a condition initially considered to be benign, including peritoneal washings, as well as peritoneal and omental biopsies, as accurate staging is crucial in determining postoperative treatment in histologically diagnosed OC [57,60,61,62,63].

### 4.2. Borderline Ovarian Tumors

Borderline ovarian tumors (BOTs) are a class of neoplasms with intermediate biological evolution and behavior between benign and malignant tumors [64]. The surgical management of this peculiar category of ovarian tumors can be either via laparotomy or laparoscopy, according to ESGO and French National College of Obstetricians and Gynecologists (CNGOF) guidelines [63,65], with the laparoscopic approach gaining increasing popularity in recent years [66]. Similarly to the laparoscopic approach of suspicious-for-malignancy ovarian masses, the main concern and adverse event is the intraperitoneal rupture of the tumor, which increases the risk of recurrence in the future but is generally considered safe in the hands of an experienced gynecologic oncologist [67,68]. Unilateral or bilateral salpingo-oophorectomy is the standard practice, depending on the stage of the disease, which is definitively determined by complete surgical staging, consisting of detailed examination of the abdominal cavity, infracolic omentectomy, peritoneal washings, and diaphragmatic, paracolic, and pelvic peritoneal biopsies, and at least inspection, if not excision, of the appendix in the case of mucinous borderline tumors [65,69]. The surgical approach is altered in cases of fertility-sparing operations for BOTs, which is often requested given the high incidence of this type of tumor in women of childbearing age. In that case, LPS is feasible for patients with stage IA-IB serous BOTs, where laparoscopic cystectomy without rupture of the tumor can be performed, as well as for patients with stage IA-IB mucinous BOTs, for which adnexectomy is required, either by an open or laparoscopic route. In both cases, hysterectomy can be omitted, while laparoscopic staging, as described above, should follow [65,69]. According to CNFOG guidelines, fertility-sparing surgery may also be an option in stage II-III BOTs, where the uterus and the contralateral ovary can be preserved, only if they are macroscopically healthy. Laparoscopy can be individually discussed with these patients, whose management should definitely be decided by multidisciplinary tumor boards [65].

### 4.3. Early-Stage Ovarian Cancer

The use of laparoscopy in the surgical treatment of early-stage OC, regarding oncological safety and feasibility, has been endorsed by a plethora of studies concluding that laparoscopy can be considered as safe and effective as laparotomy, with additional benefits and fewer perioperative complications [70]. A lower estimated blood loss, lower rates of perioperative transfusion, shorter hospital stay, faster return to normal activity, improved postoperative patient performance status, and even shorter intervals to adjuvant chemotherapy have been reported for patients with early-stage OC surgically treated with the laparoscopic approach compared to laparotomy [71,72,73,74].

However, the majority of studies reporting on the outcomes of early-stage OC patients treated with laparoscopy are retrospective [72,75,76] and thus of low quality. Published systematic reviews and meta-analyses that compared operative and survival outcomes between the laparoscopic and laparotomic approaches reported comparative results with no statistically significant difference between the two approaches [77,78,79,80,81], but they are also based on retrospective data with a low quality of evidence due to the prominent lack of prospective randomized studies. Under this scope, the consensus is that the laparoscopic approach in early-stage OC is feasible, but the lack of strong, grade I evidence led the majority of published national and international guidelines to officially recommend, until today, that surgical management and staging should be performed mainly by laparotomy. The European Society of Gynecologic Oncology (ESGO) states that the surgical treatment of stage I OC, as well as restaging surgery, could be performed laparoscopically by an experienced gynecological oncologist that has been appropriately trained to perform adequate surgical evaluation and staging laparoscopically [63], while both the International Society of Gynecologic Endoscopy (ISGE) and the American Society of Clinical Oncology recommend in their guidelines that laparoscopic surgical treatment of early-stage OC should be offered to meticulously selected patients and be performed by gynecologic oncologists with expertise in laparoscopy [17,61].

The laparoscopic surgical treatment of early-stage OC includes the visual assessment of the entire peritoneal cavity, aspiration of ascites/peritoneal washings for cytology prior to the manipulations of the tumor, total hysterectomy with bilateral salpingo-oophorectomy, infracolic (at least) omentectomy, bilateral pelvic and para-aortic lymph node dissection up to the level of the left renal vein, and biopsy of any suspicious areas for complete staging (or blind peritoneal biopsies in the case of no visible suspicious implants) [17,61,63]. It is of utmost importance to avoid intraoperative rupture of a yet-unruptured adnexal mass, especially in the case of suspected mucinous tumors, for which evaluation and visual examination of the appendix is at least required, with appendicectomy being the safer option [63]. Fertility-preserving surgery, with laparoscopic unilateral salpingo-oophorectomy and omission of total hysterectomy, but with complete surgical staging as previously described, should be offered to select premenopausal patients with stage I OC desiring fertility or the preservation of ovarian function, after discussion with the patient about the potential future risk of recurrence [17,61,63].

### 4.4. Advanced and Recurrent Ovarian Cancer

After the introduction of the Fagotti score by Anna Fagotti in 2006 [82,83], the role of diagnostic laparoscopy for diagnosing resectability and evaluating the feasibility of radical cytoreductive surgery in women with advanced OC has widely expanded. Staging laparoscopy is now considered a safe and effective method to guide treatment planning in patients with radiological findings suggestive of advanced OC [84]. The direct inspection of the peritoneal cavity and the mapping of disease dissemination allow for the more accurate prediction of operability and careful selection of cases suitable for primary debulking surgery, which is of great importance since the presence of residual disease after primary surgery is a significant adverse prognostic factor [85,86,87]. Using this approach, futile laparotomies or suboptimal debulking surgeries leading to residual disease > 1 cm can be avoided, although in clinical practice, there is a tendency to consider any residue, even that of <1 cm, as still suboptimal due to the worse prognosis. Additionally, LPS is associated with fewer perioperative complications that the patient may face after such radical operations, with minimal adverse events and reduced morbidity rates [88,89]. However, some patients may still have suboptimal primary surgery with residual disease > 1 cm, despite laparoscopy predicting optimal debulking, since the effectiveness of this procedure and, therefore, the accuracy of the prediction models depend on the experience of the surgeon performing the laparoscopy, as well as the primary debulking surgery [17,90]. Another issue that affects the oncologic result of residual disease is the optimal number of neoadjuvant chemotherapy (NACT) cycles. While traditional protocols recommend interval debulking surgery after 3–4 cycles, emerging evidence suggests that extending NACT beyond six cycles may be warranted in select patients to maximize the probability of achieving complete cytoreduction (CC0)—the strongest independent prognostic factor for survival. Recent studies demonstrate that patients achieving CC0 after ≥7 NACT cycles experience significantly improved overall survival compared to those with residual disease after six cycles, without increased chemotherapy-related toxicity. However, patient selection through diagnostic laparoscopy remains critical to identify candidates most likely to benefit from this strategy [91,92]. Still, diagnostic laparoscopy has been established as a useful tool in the diagnostic and therapeutic management of advanced-stage OC, used not to improve survival rates but to reduce perioperative morbidity by correctly identifying inoperable cases and avoiding suboptimal debulking operations [17,57,88,89,90].

Another significant benefit of diagnostic laparoscopy is the acquisition of tissue for pathological examination and the identification of the histological subtype of OC, which enables the tailoring of neoadjuvant chemotherapy for cases not suitable for primary debulking surgery [70,84]. This is particularly important given that many patients develop early recurrences or demonstrate platinum resistance despite standard platinum-based regimens. The tissue obtained through laparoscopy not only allows for molecular profiling but also provides the opportunity for ex vivo assessment of chemotherapy response, an emerging approach that could enhance patient outcomes by identifying optimal therapeutic strategies in the neoadjuvant setting prior to interval debulking surgery [93].

In these cases, a minimally invasive surgical approach also allows for the earlier administration of chemotherapy, as the recovery period that is usually required after LPS is shorter compared to laparotomy [86]. Using the same principles, laparoscopy could also be useful for predicting inoperability in patients scheduled for interval debulking surgery [94].

Finally, the role of laparoscopy in advanced OC could be further expanded in the setting of interval debulking surgery for patients who have received neoadjuvant chemotherapy. Using the same principles, laparoscopy could also be useful for predicting inoperability in patients scheduled for interval debulking surgery [94]. Additionally, recent studies have shown that laparoscopy could be implemented as an alternative approach to laparotomy for interval debulking surgery, supporting that in carefully selected patients with low-burden disease and complete radiological response, complete laparoscopic interval debulking surgery could achieve similar survival outcomes to open laparotomy [17,95,96,97,98]. However, since strong evidence is yet to be acquired, future research is required to validate these remarks and establish the use of laparoscopy in interval debulking surgery.

In the case of recurrent OC, the role of laparoscopy remains rather controversial. Although there are studies suggesting that the laparoscopic approach could be useful in terms of preoperative evaluation of the extent of disease and the feasibility of cytoreduction, the level of evidence is currently low [70,89,99]. At the same time, secondary laparoscopic cytoreduction in recurrent OC is already offered to carefully selected patients who have previously undergone comprehensive evaluation of their clinical condition, performance status, previous treatments, and the extent of disease recurrence [100]. While no significant differences in survival outcomes are reported in the few existing studies [79,100,101], there is no consistent data currently available to identify patients eligible for laparoscopic surgical management of recurrences, and the decision is often left to surgeons’ discretion [89].

### 4.5. Non-Epithelial Ovarian Cancer

Non-epithelial ovarian tumors account for approximately 10% of all ovarian cancers and are mainly diagnosed in young women of reproductive age, with notable differences not only in epidemiology but also in the diagnostic and therapeutic approach compared to epithelial OC [102,103]. Traditionally, non-epithelial OCs are surgically treated with laparotomy according to the guidelines of ESGO and the European Society of Medical Oncology (ESMO). Laparotomy is usually preferred in order to avoid tumor rupture and is traditionally considered a more appropriate approach, as these tumors are often solid and of large size [104,105]. However, laparoscopic or even robotic approaches are also acceptable in select cases and with specific requirements, including the training of the operating doctor in laparoscopic gynecological oncology, the application of safety measures to avoid rupture of the tumor, and the complete exploration of the peritoneal cavity [69,106]. Regarding the surgical technique, laparoscopic oophorectomy is preferred to cystectomy, while adequate surgical staging is required, including at least peritoneal fluid sampling for cytology, examination of peritoneal surfaces, a biopsy of the diaphragmatic peritoneum, paracolic gutters, and pelvic peritoneum, a large biopsy of the omentum, and inspection of the contralateral ovary with a biopsy of areas with suspicious appearance [69,106,107].

#### Key Points—Summary of Current Clinical Practice

Laparoscopy is gradually gaining more recognition as a surgical approach in the management of OC, a malignancy which is traditionally treated with laparotomy. For suspicious adnexal masses, laparoscopic en bloc oophorectomy or adnexectomy of the impacted adnexa is considered an oncologically safe alternative approach under the condition that appropriate measures, such as the use of laparoscopic endobags, are applied by experienced gynecological oncologists, to avoid the dissemination of potential malignancy. Likewise, BOTs can also be treated laparoscopically, even in cases where fertility-sparing surgery is offered (stage IA and IB serous and mucinous BOTs), and any additional required surgical maneuvers, including omentectomy and appendicectomy, can also be performed laparoscopically. For both suspicious ovarian masses and BOTs treated with a laparoscopic approach, adequate staging, consisting at least of peritoneal washings and peritoneal and omental biopsies, should be diligently performed. Regarding the surgical treatment of early-stage OC, which usually consists of the aspiration of ascites/peritoneal washings for cytology prior to manipulations of the tumor, total hysterectomy with bilateral salpingo-oophorectomy, infracolic (at least) omentectomy, and bilateral pelvic and para-aortic lymph node dissection up to the level of the left renal vein, the abdominal approach remains the gold standard. The lack of high-quality evidence about the oncological safety of LPS has led to a restricted offer of this approach to very carefully selected patients with early-stage OC (including non-epithelial OC) who are being treated by very experienced and appropriately trained gynecological oncologists. The role of laparoscopy is very limited and mainly experimental in the cases of advanced-stage and recurrent OC, with the exception of staging diagnostic laparoscopy, which is recognized as an oncologically safe procedure for patients with suspected advanced OC, as it enables the accurate staging and selection of patients eligible for optimal debulking surgery and the acquisition of tissue specimens for the confirmation of the subtype of OC and tailoring of systemic therapies.

## 5. Vulvar Cancer

Laparoscopy was not included in the surgical management of vulvar cancer until recently, when laparoscopy-assisted sentinel lymph node (SLN) biopsy was introduced as part of its surgical treatment [108]. Intraoperative lymphatic mapping for vulvar cancer was first reported by Levenback in 1994, who used isosulfan blue dye to detect the inguinal sentinel lymph nodes [109]. However, as a great variety of studies throughout the years have supported the superiority of ICG as a tracer for SLN identification in other types of gynecological cancers, such as cervical and EC [110,111], SLN biopsy using ICG with near-infrared fluorescence imaging has emerged as a safe and effective technique in patients with vulvar cancer [112]. Although not performed laparoscopically, the detection of SLN when ICG is used requires a near-infrared fluorescence imaging system [113] and is frequently performed with the use of a video-laparoscopic system with a fluorescence imaging system (which is routinely used for SLN mapping in endometrial and cervical cancer) in order to visualize the colored node, while it has been demonstrated that ICG should be combined with Tc99-nanocolloid when available, as it appears to be the tracer combination with the best performance in the intraoperative detection of SLN [114]. According to the ESGO guidelines for the management of vulvar cancer, the use of Tc99-nanocolloid is mandatory when SLN biopsy is indicated, and combining it with ICG is recommended [115].

Apart from SLN detection, LPS is gradually expanding in the surgical management of vulvar cancer, as laparoscopic minimally invasive inguinal lymphadenectomy is a relatively new, safe, and effective technique that is gaining in interest among gynecologic oncologists, accompanied by shorter hospital stays and a lower risk of complications compared to open lymphadenectomy [116,117,118]. There are currently two well-described surgical approaches for video endoscopic inguinal lymph node dissection, the limb subcutaneous surgical approach (VEIL-L), and the hypogastric subcutaneous approach (VEIL-H), with comparable perioperative results [116,119]. Moreover, another approach of laparoscopic inguinofemoral lymph node dissection was recently reported by Ding et al., who described the gasless single-port laparoscopic inguinal lymphadenectomy through vulva incision and assessed the short-term efficacy of this new technique, which decreases the difficulty of operation by reducing operation time and blood loss compared to classic laparoscopic inguinal lymphadenectomy [120]. Finally, laparoscopy can also be applied to the surgical management of vulvar cancer when pelvic lymph node dissection is indicated. Although the role of pelvic systematic lymphadenectomy remains ambiguous in the staging and treatment of vulvar cancer, it can be considered for a subcategory of patients at high risk for pelvic nodal involvement, allowing for the omission of pelvic radiotherapy when the excised lymph nodes are negative for metastasis [121,122], while the dissection of enlarged pelvic lymph nodes can be discussed and offered before (chemo)radiotherapy in cases of pelvic lymph node recurrence [115].

### Key Points—Summary of Current Clinical Practice

The role of laparoscopy in the treatment of vulvar cancer is very limited, as its application has only been recently explored in this malignancy. For patients requiring inguinal lymphadenectomy, the minimally invasive approach, either VEIL-L or VEIL-H, is an oncologically acceptable and safe alternative to open lymph node dissection, which remains the recommended procedure. Moreover, when indicated, additional pelvic lymphadenectomy can also be performed laparoscopically, although the necessity and the effectiveness of the procedure itself are disputed in the setting of vulvar cancer.

## 6. Breast Cancer

Sentinel lymph node biopsy has been established as the gold standard for the surgical assessment and staging of axillary lymph nodes and has widely replaced axillary lymphadenectomy, which is nowadays indicated only in a small proportion of patients with breast cancer [123]. The technique of SLN detection in breast cancer was first applied with the use of isosulfan blue, but following the introduction of radioisotopes as tracers, dual mapping with the use of a combination of tracers was vastly preferred, since it significantly increased the sensitivity and the SLN detection rates [124]. The identification of SLN using near-infrared fluorescence laparoscopy with ICG as a tracer, successfully established in gynecologic oncology, has been successfully adapted for breast cancer axillary staging, as it ensures a good visualization of lymphatic vessels, the precise excision of the SLN, and detection rates comparable to other tracers, with further improvement when combined with radiocolloid [125,126]. Although the liposuction of axillary fat was considered a necessary step that should precede the trocar placement and the excision of the SLN, creating more operating space in the axilla and enabling a clear display of the lymph nodes, there are new techniques described for axillary SLN biopsy where liposuction can be omitted in an effort to decrease and eradicate its potential complications [127]. Single-port non-liposuction near-infrared laparoscopy with ICG injection for axillary SLN biopsy was assessed by Yao et al., who compared the technique with standard open SLN detection using near-infrared imaging, concluded that it is non-inferior to the open approach and may be an appropriate alternative option for patients with early breast cancer who qualify for breast-conserving surgery and desire a better cosmetic outcome with fewer incisions [128]. Following the same philosophy, and for patients undergoing breast-preserving operations for early breast cancer, non-liposuction laparoscopic axillary SLN excision can also be performed through a periareolar incision [129]. Using the same techniques described above, axillary lymphadenectomy can also be performed laparoscopically when required, using a single or multiple ports, with or even without liposuction, with comparable results regarding safety and efficacy to open axillary lymphadenectomy, with the potential advantage of the earlier and easier mobilization of the upper limb, facilitated by the absence of an axillary scar, which also leads to a superior aesthetic outcome [130,131].

Breast surgery was traditionally thought to be feasible solely by the open surgical approach; however, the rapid evolution of laparoscopy has led to the development of minimally invasive techniques not only for surgical staging but also for breast surgical procedures, both radical and breast-conserving, in patients with breast cancer. Laparoscopic/endoscopic breast-conserving skin-sparing quadrantectomy, with single or multiple incisions, accompanied or not by axillary SLN biopsy or even systematic lymphadenectomy, tailored according to each patient’s needs and characteristics, can be offered to carefully selected patients and combines cosmetic improvement and oncological safety [132,133,134]. For cases where breast-conserving surgery is not an option, more radical breast surgical procedures may also be performed via a laparoscopic approach, including complex operations such as nipple-sparing mastectomy combined with prosthesis breast reconstruction and laparoscopic radical mastectomy with omental breast reconstruction, without statistically significant differences in complication rates and survival outcomes when compared with the open route [135,136]. As minimally invasive surgery evolves, a greater variety of breast surgeries may be feasible using laparoscopic techniques, but further studies will be required to validate the safety of this approach. To conclude, laparoscopy can also contribute to diagnostic evaluation or even the application of prophylactic measures for patients with breast cancer. Laparoscopy can confirm the presence of peritoneal dissemination of breast cancer in patients with advanced-stage disease and suspected distant peritoneal metastases with inconclusive imaging, while laparoscopic prophylactic salpingo-oophorectomy can be offered in patients with breast cancer and BRCA gene mutations as a combined procedure with breast oncological and/or reconstructive surgery [137,138].

### Key Points—Summary of Current Clinical Practice

The role of laparoscopy in the management of breast cancer is mainly limited to axillary surgical staging. While laparoscopic SLN biopsy is feasible and may be offered to select patients seeking optimal cosmetic outcomes, open surgery remains the standard approach. Regarding breast surgery, laparoscopic/endoscopic breast-conserving skin-sparing quadrantectomy or even mastectomy can be offered to select patients and requires validation through prospective studies demonstrating oncological safety, cost-effectiveness, and reproducibility across centers before routine adoption.

## 7. Laparoscopic Techniques with Special Interest

Although various innovative applications of laparoscopy in gynecological malignancies have been described, special attention should be paid to a rather recent laparoscopic advancement, the retroperitoneal laparoscopic para-aortic lymph node dissection. This technique was first described by Dargent et al. in 2000 for the surgical staging of cervical cancer and has since been gradually implemented into the surgical management of other gynecological cancers [108]. Compared with the transperitoneal approach, retroperitoneal laparoscopic para-aortic lymphadenectomy is characterized by similar oncological safety and efficacy [109] and has significant advantages, such as an improved surgical field, reduction in bowel injuries, adhesion formation and intraoperative complications in general, avoidance of the Trendelenburg position during surgery, a reduction in operational time, easier access to upper para-aortic lymph nodes, and subsequent increased mean number of obtained lymph nodes [111,112,113]. The retroperitoneal laparoscopic approach can be applied for the surgical staging of early-stage OC, high-risk EC, and especially cervical cancer, where its use has increased, as surgical staging in advanced-stage disease is gaining interest and acceptance [112]. Future studies are expected to highlight the benefits, expand the indications of application, and further establish the use of this surgical approach in gynecological malignancies.

## 8. Conclusions

LPS is increasingly utilized in the diagnostic and therapeutic management of gynecological cancers. For EC, the laparoscopic approach is considered the gold standard for the treatment of early-stage disease, as well as for surgical staging with the use of SLN mapping. The use of laparoscopy in the treatment of early-stage cervical cancer was significantly decreased in recent years and is reserved for carefully selected cases but is gradually gaining interest in the surgical staging of women with locally advanced disease. OC could be surgically treated with laparoscopy in its early stages by an experienced gynecologic oncologist, as an acceptable alternative to laparotomy. Finally, although laparoscopy was not traditionally considered a feasible approach for the treatment of vulvar and breast cancer, novel techniques are continuously arising and allow the establishment of the use of LPS in these gynecologic malignancies. Future research will further elucidate and potentially expand the role of laparoscopy in gynecological cancer.

## Data Availability

Not applicable.
